# Augmented reality-assisted versus conventional total hip arthroplasty: a systematic review and meta-analysis

**DOI:** 10.1186/s13018-023-04421-0

**Published:** 2023-12-02

**Authors:** Shilong Su, Ruideng Wang, Zhengyang Chen, Fang Zhou, Yunqing Zhang

**Affiliations:** 1 Department of Orthopedics, Peking University Third Hospital, No.49 North Garden Road. Haidian, Beijing, 100191 China; 2 Engineering Research Center of Bone and Joint Precision Medicine, Peking University Third Hospital, No.49 North Garden Road. Haidian, Beijing, 100191 China; 3 Department of Orthopedics, The First Hospital of Changsha, No.311 Yingpan Road, Changsha, 410005 Hunan Province China

**Keywords:** Augmented reality, Extended reality, Navigation, Total hip arthroplasty, Meta-analysis

## Abstract

**Background:**

Extended reality (XR), including virtual reality, augmented reality (AR), and mixed reality, has been used to help achieve accurate acetabular cup placement in total hip arthroplasty (THA). This study aimed to compare the differences between XR-assisted and conventional THA.

**Methods:**

In this systematic review and meta-analysis, electronic databases including PubMed, Embase, Web of Science, Cochrane Central Register of Controlled Trials (CENTRAL), and clinicaltrials.gov were searched for studies from inception to September 10, 2023. The outcomes were accuracy of inclination and anteversion, duration of surgery, and intraoperative blood loss. Meta-analysis was performed using Review Manager 5.4 software.

**Results:**

A total of five studies with 396 patients were included in our study. The pooled results indicated AR-assisted THA had better accuracy of inclination and anteversion than conventional THA (SMD = − 0.51, 95% CI [− 0.96 to − 0.07], *P* = 0.02; SMD = − 0.96, 95% CI [− 1.19 to − 0.72], *P* < 0.00001), but duration of surgery and intraoperative blood loss were similar in the two groups.

**Conclusion:**

This systematic review and meta-analysis found that AR-assisted THA had better accuracy of inclination and anteversion than conventional THA, but the duration of surgery and intraoperative blood loss were similar in the two groups. Based on the pooled results, we suggested that AR can provide more precise acetabular cup placement than conventional methods in THA.

**Supplementary Information:**

The online version contains supplementary material available at 10.1186/s13018-023-04421-0.

## Introduction

Total hip arthroplasty (THA) is the main and effective surgical method for the treatment of advanced hip osteoarthritis, osteonecrosis, and rheumatoid arthritis [1]. The acetabular cup position is very important for maximizing the hip range of motion and minimizing impingement, dislocation, liner fracture, and long-term wear [2–4]. Good acetabular anteversion and inclination angle is the key to the success of THA. Due to pelvic movement, complex anatomy, and varying surgeon experience, it is difficult and error-prone to use conventional surgical techniques to accurately and consistently locate the acetabular cup [5]. Cup malposition is associated with multiple postoperative complications, such as impingement, dislocation, liner fracture, and the need for revision surgery, burdening the patient [6–8]. Therefore, to minimize the risk of dislocation, the "safe zone" of the position of the acetabular prosthesis described by Lewinnek et al. has been used as a guide [9].

Many techniques can help achieve accurate component position, including intraoperative radiographs [10], fluoroscopy [11], computer-assisted navigation systems [12], and robotics [13]. However, these solutions proposed have substantial limitations, including X-ray exposure to the patient and personnel, lack of accuracy due to parallax or operator error with fluoroscopy, cost, or change in pelvis position during the operation. To resolve these issues, we are witnessing increasing use of augmented reality (AR), virtual reality (VR), and new mixed reality (MR), which include both AR and VR, to assist in THA. VR technology generally uses a headset, blocking out visual stimuli from the real world. AR allows users to see the real world but overlays virtual elements. MR combines the two, including aspects of both the real and virtual worlds [14, 15]. Extended reality (XR) is the umbrella term that refers to these three different types of technology [16]. Generally, XR-assisted THA provides accurate and reproducible component positioning and balancing of soft tissue. These benefits may contribute to longer implant survival and a reduced need for revision surgery [17–19]. However, some scholars do not agree with these results [20].

Both XR-assisted and conventional methods of THA have been compared in many clinical trials; however, most of these studies have small sample sizes. XR-assisted THA requires a larger operating space, wider exposure, and longer operation time, which may increase the probability of postoperative infection. The choice between XR-assisted and conventional approaches for THA remains controversial. To our knowledge, no systematic review and meta-analysis has compared the safety and efficacy between XR-assisted and conventional methods of THA. Thus, this systematic review and meta-analysis was designed to compare the differences between XR-assisted and conventional THA, to gain some theoretical insights that may guide clinical practice.

## Methods

This systematic review and meta-analysis was reported according to the Preferred Reporting Items for Systematic Reviews and Meta-Analyses (PRISMA) [21]. The protocol was registered in the International Prospective Register of Systematic Reviews (PROSPERO) (CRD42022364486).

### Search strategy and selection criteria

We searched PubMed, Embase, Cochrane Central Register of Controlled Trials (CENTRAL), Web of Science, and clinicaltrials.gov electronic databases from inception to September 10, 2023, with restriction to the English language. We used the following search terms in each database: (virtual reality OR augmented reality OR mixed reality) AND total hip arthroplasty. To achieve the highest sensitivity, we used a combination of keywords and indexed terms (e.g., PubMed Medical Subject Headings). We also examined the reference lists of each comparative study and reviews to identify additional relevant studies. The detailed search strategy is available in the Supplement.

The criteria for inclusion were research articles studying VR-, AR-, or MR-assisted compared to conventional THA and reporting on the accuracy of inclination and anteversion, duration of surgery, and intraoperative blood loss. Two investigators (SS and RW) independently screened all identified articles and considered the potential eligibility of each of the titles and abstracts. Full-text articles were obtained unless both reviewers decided that an abstract was ineligible. Disagreements between reviewers were discussed and resolved by consensus.

### Data extraction and quality assessment

Two investigators (SS and RW) independently extracted data from all included studies using a data extraction form. Any disagreements between them were solved by a discussion. The included studies were evaluated for authors, year of publication, country, study design, number of patients, age, sex, surgical approach, type of XR, the accuracy of inclination and anteversion, duration of surgery, and intraoperative blood loss. If data were not presented in the original article, corresponding authors were contacted to acquire the missing data, although no responses were received.

Two investigators (SS and RW) evaluated the quality of the included studies independently, utilizing the Risk of Bias tool of the Cochrane Library [22, 23]. Funnel plots were used to assess publication bias for any of the outcomes, and a publication bias was considered present if an asymmetry in the funnel plot was found. Any disagreements between the two reviewers were resolved by a discussion.

### Statistical analysis

For continuous outcomes, including the accuracy of inclination and anteversion, duration of surgery, and intraoperative blood loss, the mean difference (MD) and associated 95% confidence interval (CI) were used to perform estimates for each study. If different measurement methods or units were used for the same index and the mean values were significantly different, standardized mean difference (SMD) with 95% CI was used. We utilized the random-effect or fixed-effect model to analyze the pooled results, respectively, when significant heterogeneity (*P* < 0.10; *I*^2^ > 50%) appeared or not. The sensitivity analysis was performed to evaluate the reliability of the pooled results by removing some studies from analyzed studies in each analysis, and subgroup analysis was conducted to obtain more specific conclusions. All statistical analyses were performed using the Review Manager software (version 5.4, Cochrane Collaboration, Oxford, UK). A *P*-value < 0.05 was considered significant.

## Results

### Study identification

**Fig. 1:**
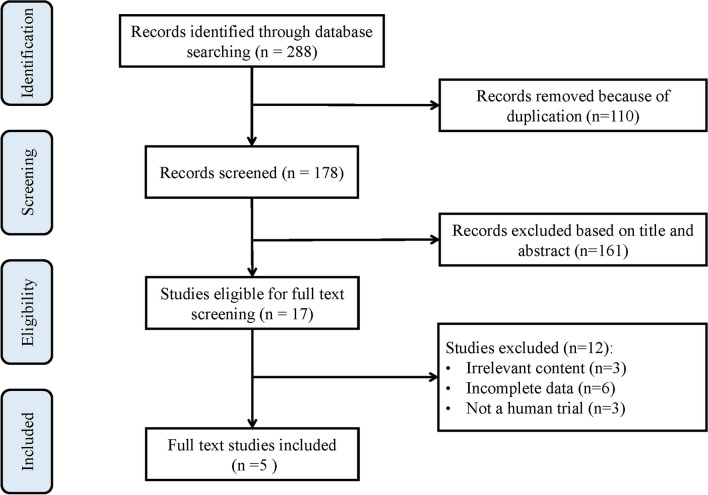
PRISMA (Preferred Reporting Items for Systemic Meta-Analyses) flowchart for study identification process

As shown in Fig. 1, there were 219 studies yielded from the five electronic databases. After removing 110 duplicates, 178 studies remained. After screening the titles and abstracts and reading full texts, 173 studies were excluded. Thus, five studies published from 2019 to 2023 were finally included in the study, including three randomized, controlled trials (RCTs) [20, 24, 25] and two retrospective cohort studies [26, 27].

### Study characteristics and quality assessment

**Fig. 2:**
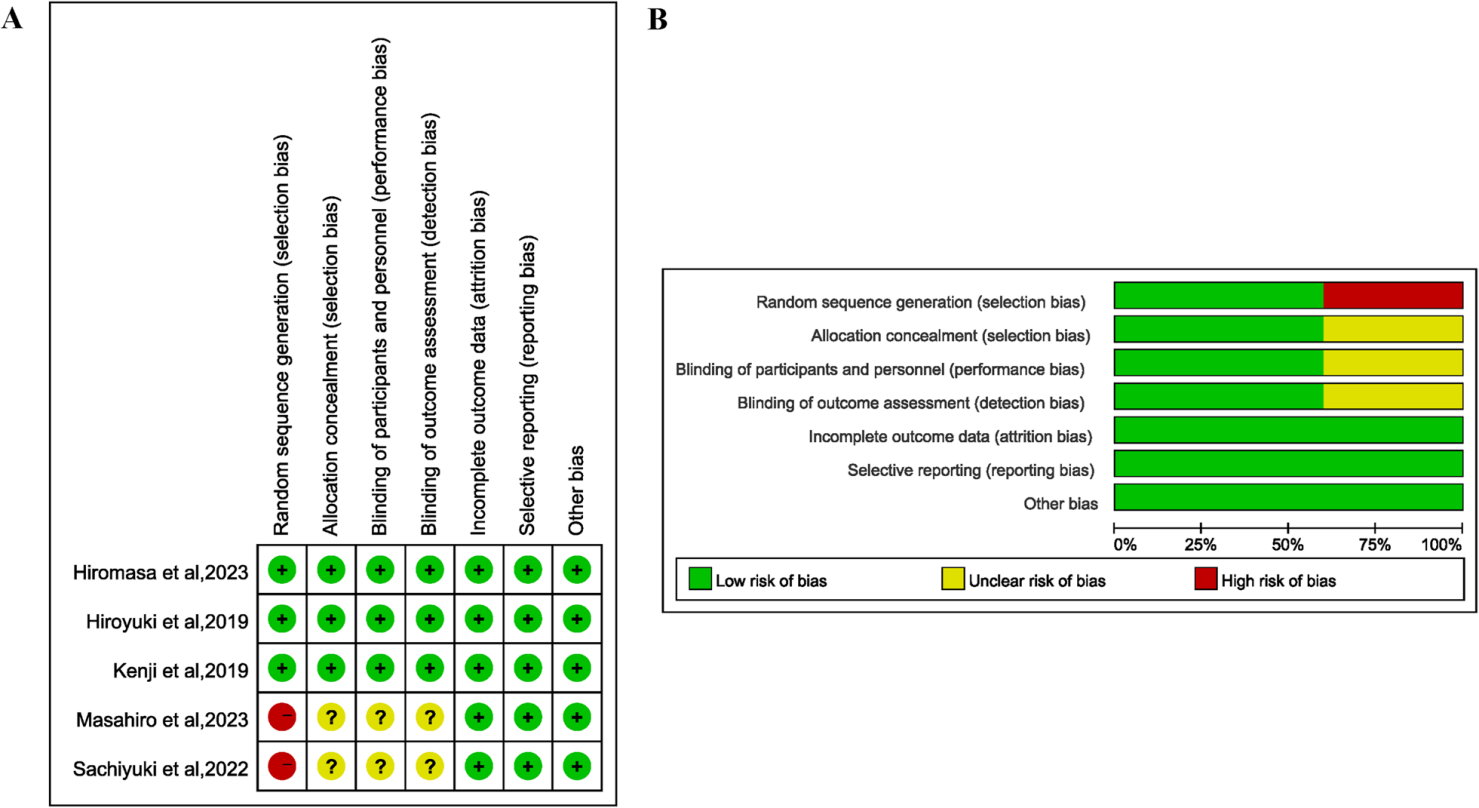
Quality evaluation of included studies utilizing the Risk of Bias tool of the Cochrane Library. **A** Judgments of authors about each risk of bias item for each included study and **B** about each risk of bias item presented as percentages across all included studies

A total of five studies enrolled 396 patients with 200 undergoing AR-assisted THA and 196 undergoing conventional THA. The type of XR used only included AR, without VR and MR. The detailed data of the included studies are summarized in Table 1. The risk of bias was low for most of the domains. Two studies were rated to have high risk due to the absence of randomization [26, 27]. The result of the risk of bias is summarized in Fig. 2. There was no evidence of publication bias after the assessment of the funnel plots (Additional file 1: Figures S1, S2, S3, and S4 in the Supplement).

**Table 1:** Characteristics of included studies

Author	Country	Study design	Participants	*N* (I/C)	Age (Mean ± SD, years) (I/C)	Female, % (I/C)	Surgical approach	Intervention	Comparator
Hiromasa et al. [24]	Japan	RCT	Patients undergoing primary THA	36/36	65 ± 11/66 ± 10	83.33/83.33	Standard posterior	AR-based portable hip navigation system	Conventional mechanical guide
Hiroyuki et al. [20]	Japan	RCT	Patients undergoing THA	22/19	65 ± 11/67 ± 12	86.36/89.47	Modified Watson-Jones	AR-based portable navigation system	Conventional freehand technique with a mechanical alignment guide
Kenji et al. [25]	Japan	RCT	Patients undergoing unilateral primary THA	62/64	67 ± 10/69 ± 10	80.65/85.94	Modified Watson-Jones	AR-based portable navigation system	Accelerometer-based portable navigation system
Masahiro et al. [26]	Japan	Retrospective cohort study	Patients underwent THA	35/35	64.4 ± 14.7/67.1 ± 10.4	77.14/82.86	NA	AR-based navigation system	Accelerometer-based handheld surgical navigation system
Sachiyuki et al. [27]	Japan	Retrospective cohort study	Patients underwent primary THA	45/42	66 ± 9/62 ± 11	84.44/80.95	Modified Watson-Jones	AR-based portable navigation system	Accelerometer-based portable navigation system

*N* The number of participants, *I* Intervention, *C* Control, *SD* Mean difference, *RCT* Randomized clinical trial, *THA* Total hip arthroplasty, *AR* Augmented reality, and *NA* Not available

### Accuracy of inclination

**Fig. 3:**
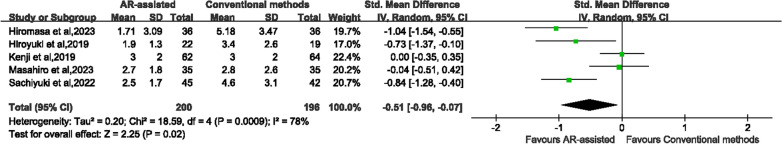
Forest plot showing accuracy of inclination between two groups

All five studies reported accuracy of inclination and were included in the meta-analysis [20, 24–27]. The results showed that AR-assisted THA had better accuracy of inclination than conventional THA (SMD = − 0.51, 95% CI [− 0.96 to − 0.07], *P* = 0.02) (Fig. 3). Because of the existence of heterogeneity (*P* = 00009, *I*^2^ = 78%), the sensitivity analysis was performed. However, we found that heterogeneity was still high after excluding studies one by one. We conducted subgroup analyses by dividing the studies into the RCTs subgroup and the retrospective cohort studies subgroup. In the RCTs subgroup, due to the significant heterogeneity (*P* = 0.002, *I*^2^ = 84%), the sensitivity analysis was performed and indicated that the heterogeneity came from one study [25]. Therefore, we performed analysis again after removing the study, and the results revealed that AR-assisted THA had better accuracy of inclination than conventional THA (SMD = − 0.93, 95% CI [− 1.32 to − 0.54], *P* < 0.00001) in the RCTs subgroup, while no significant difference in the retrospective cohort studies subgroup (SMD = − 0.45, 95% CI [− 1.23 to 0.33], *P* = 0.26) (Additional file 1: Figure S5). It is worth noting that after excluding this study [25], only two RCTs in the RCTs subgroup were included in the analysis.

### Accuracy of anteversion

**Fig. 4:**
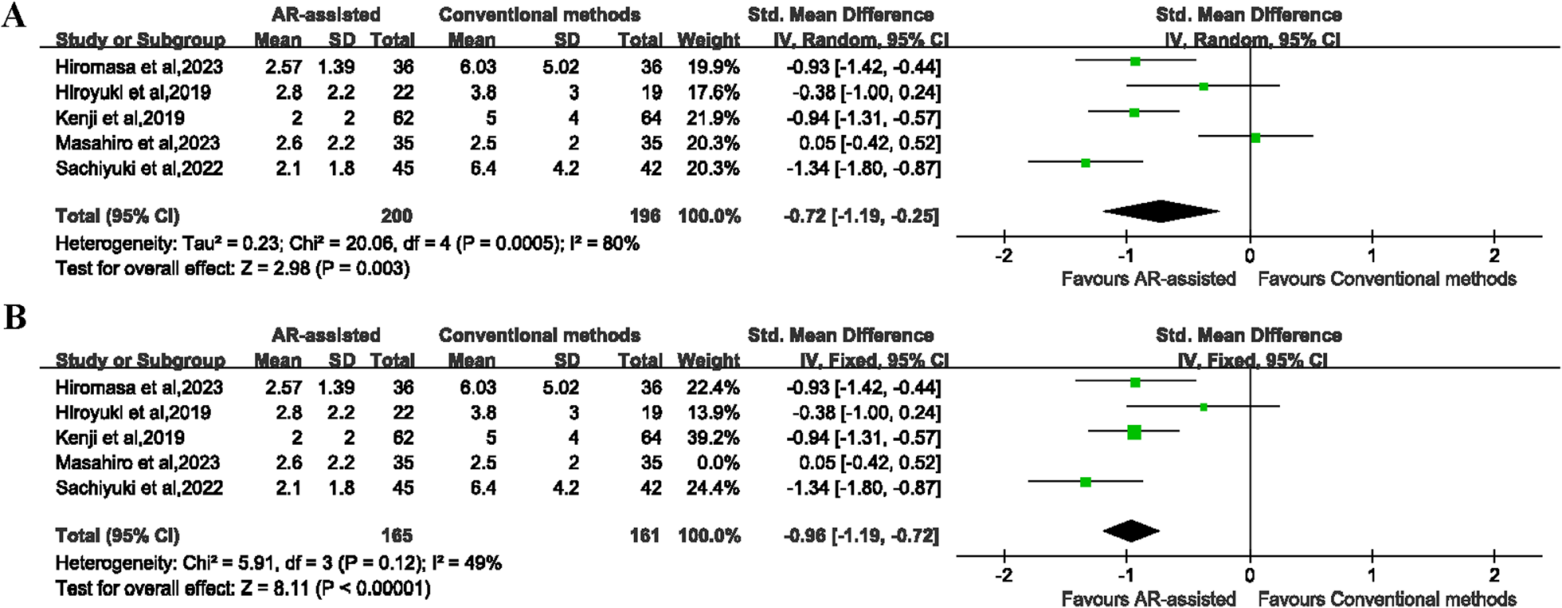
**A** Forest plot showing accuracy of anteversion between two groups. **B** Forest plot showing accuracy of anteversion after removing one study between two groups

All five studies reported accuracy of anteversion and were included in the meta-analysis [20, 24–27]. The pooled results showed that AR-assisted THA had better accuracy of anteversion than conventional THA (SMD = − 0.72, 95% CI [− 1.19 to − 0.25], *P* = 0.003) (Fig. 4A). Due to the heterogeneity (*P* = 0.0005, *I*^2^ = 80%), the sensitivity analysis was performed and indicated that the heterogeneity came from one study [26]. Therefore, we performed analysis again after removing the study, and the results also revealed that AR-assisted THA had better accuracy of anteversion than conventional THA (SMD = − 0.96, 95% CI [− 1.19 to − 0.72], *P* < 0.00001) (Fig. 4B). The subgroup analyses showed that AR-assisted THA had better accuracy of anteversion than conventional THA (SMD = − 0.82, 95% CI [− 1.12 to − 0.51], *P* < 0.00001) in the RCTs subgroup, while no significant difference in the retrospective cohort studies subgroup (SMD = − 0.64, 95% CI [− 2.00 to 0.71], *P* = 0.35) (Additional file 1: Figure S6).

### Duration of surgery

**Fig. 5:**
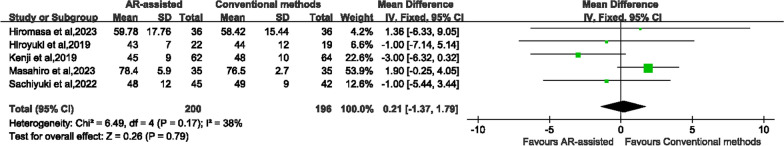
Forest plot showing duration of surgery between two groups

All five studies reported duration of surgery and were included in the meta-analysis [20, 24–27]. The results showed that no significant differences were found between the two groups (MD = 0.21, 95% CI [− 1.37 to 1.79], *P* = 0.79) (Fig. 5). The subgroup analyses also showed that there were no significant differences between the two groups in the RCTs subgroup and in the retrospective cohort studies subgroup (Additional file 1: Figure S7).

### Intraoperative blood loss

**Fig. 6:**
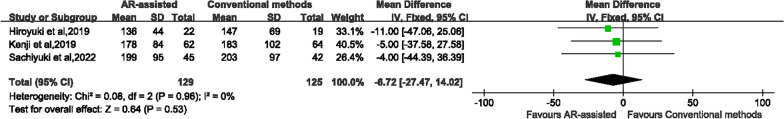
Forest plot showing intraoperative blood loss between two groups

There were three studies reporting intraoperative blood loss and included in the meta-analysis [20, 25, 27]. The pooled results showed that no significant differences were found between the two groups (MD = − 6.72, 95% CI [− 27.47 to 14.02], *P* = 0.53) (Fig. 6).

## Discussion

This systematic review and meta-analysis included five studies that assessed 396 patients and compared the accuracy of inclination and anteversion, duration of surgery, and intraoperative blood loss between AR-assisted and conventional THA groups. The pooled results revealed that AR-assisted THA had better accuracy of inclination and anteversion, but the duration of surgery and intraoperative blood loss were similar in the two groups. Our study included three RCTs and two retrospective cohort studies, and the analysis showed that the heterogeneity was high. Therefore, we carried out sensitivity analysis and subgroup analysis and confirmed the reliability of the results. To our best knowledge, this is the first systematic review and meta-analysis to show the differences between AR-assisted and conventional THA.

Accurate and appropriate acetabular cup position is one of the key factors for successful THA [2–4]. At present, the acetabular cup is mainly placed at a fixed angle, such as inclination of 40 degrees, anteversion of 15 or 20 degrees, or in the Lewinnek safe zone [28]. However, some scholars believe that the ideal cup positioning angle of each patient is different [28, 29], and it is necessary to develop a specific cup location for each patient to avoid complications after THA. This view is gradually recognized. However, there are challenges in conventional THA to achieve this goal of personalized and precise treatment. Therefore, XR-assisted THA, including AR, has been developed and received people's attention [19]. A systematic review and meta-analysis showed that XR training had better accuracy of inclination and shorter surgical duration than conventional methods in THA on the models or cadavers [30]. At present, several human clinical trials of AR-assisted THA have been published, but the reported results are controversial. So, we carried out this systematic review and meta-analysis and found that AR-assisted THA had better accuracy of inclination and anteversion than conventional THA in human trials. Since XR-assisted THA needs to expose fixed anatomical landmarks to register during surgery, people are worried that this process will lead to an increase in the duration of surgery and intraoperative blood loss. Our study found that there was no difference between AR-assisted and conventional THA in terms of duration of surgery and intraoperative blood loss. However, these studies mainly reported data on the accuracy of acetabular cup position, with little data on long-term postoperative clinical function and postoperative complications. Only one study [24] reported the Hip Disability and Osteoarthritis Outcome Scores and complications at 6 months after operation, and there was no difference between the two groups. Therefore, it is unknown whether the improvement in imaging data observed at present will bring benefits to the medium- and long-term clinical outcomes of patients after operation. High-quality long-term follow-up RCTs are also needed to verify.

Currently, surgical robots, fluoroscopy, and intraoperative computer navigation are increasingly used to improve the accuracy of THA [10–13]. However, intraoperative X-ray imaging during fluoroscopy cannot provide three-dimensional images, consumes operating room space, and increases radiation exposure to patients and surgeons. Moreover, computer navigation and surgical robots may distract surgeons from the surgical site through computer screens [31]. In addition, fluoroscopy, computer-assisted navigation, and surgical robots usually need to be equipped with large equipment and additional personnel in the operating room, which will also bring corresponding problems. XR can visually integrate data into diagnosis and surgery through a pair of glasses, such as HoloLens, without the need for additional imaging and equipment [31]. In the study, we only found AR-assisted THA, and there was no intraoperative navigation based on MR. MR is a new digital holographic imaging technology that combines the advantages of VR and AR. By introducing the real scene information into the virtual environment, the interactive feedback information cycle is established among the virtual world, the real world, and the users to enhance the authenticity of the user experience. Its key is to interact with the real world, to obtain information in time, and to interact seamlessly with the users of the real world and virtual models [14, 15]. Perhaps in the near future, the development of a portable navigation system based on MR can provide us with a more practical surgical navigation system.

Some deficiencies should be considered when generalizing the conclusion of this study. Firstly, the sample size of our study is small, and both RCTs and non-RCTs were included due to a lack of data, which adds to potential bias to this study. Secondly, because all the included studies were published in English, the language bias was difficult to avoid. Thirdly, although we have performed sensitivity and subgroup analyses, significant heterogeneity remained between studies. Further research is needed to minimize heterogeneity and improve statistical power.

## Conclusion

This systematic review and meta-analysis found that AR-assisted THA had better accuracy of inclination and anteversion than conventional THA, but the duration of surgery and intraoperative blood loss were similar in the two groups. Based on the pooled results, we suggested that AR can provide more precise acetabular cup placement than conventional methods in THA.

### Supplementary Information

**Additional file 1.** Database search algorithms, funnel plots to assess publication bias, and forest plots of the subgroup analyses.

## Data Availability

The datasets used and/or analyzed during the current study are available from the corresponding author on reasonable request.

## Abbreviations

THA: Total hip arthroplasty
AR: Augmented reality
VR: Virtual reality
MR: Mixed reality
XR: Extended reality
PRISMA: Preferred Reporting Items for Systematic Reviews and Meta-Analyses
PROSPERO: International Prospective Register of Systematic Reviews
CENTRAL: Cochrane Central Register of Controlled Trials
MD: Mean difference
CI: Confidence interval
SMD: Standardized mean difference
RCTs: Randomized, controlled trials
