# Supplementary material for: Augmented reality-assisted versus conventional total hip arthroplasty: a systematic review and meta-analysis
Source: J Orthop Surg Res. 2023 Dec 2;18:920. doi: 10.1186/s13018-023-04421-0 (PMC10693088; doi:10.1186/s13018-023-04421-0)
Supplement: Supplementary file 1 — Additional file 1. Database search algorithms, funnel plots to assess publication bias, and forest plots of the subgroup analyses. [file 13018_2023_4421_MOESM1_ESM.docx]

**Database Search Algorithms**

**PubMed**

20230910

((((((((((((((((((((("Arthroplasty, Replacement, Hip"[Mesh]) OR (Arthroplasties, Replacement, Hip[Title/Abstract])) OR (Arthroplasty, Hip Replacement[Title/Abstract])) OR (Hip Prosthesis Implantation[Title/Abstract])) OR (Hip Prosthesis Implantations[Title/Abstract])) OR (Implantation, Hip Prosthesis[Title/Abstract])) OR (Prosthesis Implantation, Hip[Title/Abstract])) OR (Hip Replacement Arthroplasty[Title/Abstract])) OR (Replacement Arthroplasties, Hip[Title/Abstract])) OR (Replacement Arthroplasty, Hip[Title/Abstract])) OR (Arthroplasties, Hip Replacement[Title/Abstract])) OR (Hip Replacement Arthroplasties[Title/Abstract])) OR (Hip Replacement, Total[Title/Abstract])) OR (Total Hip Replacement[Title/Abstract])) OR (Total Hip Arthroplasty[Title/Abstract])) OR (Arthroplasty, Total Hip[Title/Abstract])) OR (Hip Arthroplasty, Total[Title/Abstract])) OR (Total Hip Arthroplasties[Title/Abstract])) OR (Replacement, Total Hip[Title/Abstract])) OR (Total Hip Replacements[Title/Abstract])) OR (THA[Title/Abstract])) AND

(((((((((((((("Virtual Reality"[Mesh]) OR (Reality, Virtual[Title/Abstract])) OR (Virtual Reality, Educational[Title/Abstract])) OR (Educational Virtual Realities[Title/Abstract])) OR (Educational Virtual Reality[Title/Abstract])) OR (Reality, Educational Virtual[Title/Abstract])) OR (Virtual Realities, Educational[Title/Abstract])) OR (Virtual Reality, Instructional[Title/Abstract])) OR (Instructional Virtual Realities[Title/Abstract])) OR (Instructional Virtual Reality[Title/Abstract])) OR (Realities, Instructional Virtual[Title/Abstract])) OR (Reality, Instructional Virtual[Title/Abstract])) OR (Virtual Realities, Instructional[Title/Abstract])) OR (((((((("Augmented Reality"[Mesh]) OR (Augmented Realities[Title/Abstract])) OR (Realities, Augmented[Title/Abstract])) OR (Reality, Augmented[Title/Abstract])) OR (Mixed Reality[Title/Abstract])) OR (Mixed Realities[Title/Abstract])) OR (Realities, Mixed[Title/Abstract])) OR (Reality, Mixed[Title/Abstract])))

**EMBASE**

20230910

('arthroplasty, replacement, hip':ab,ti OR 'arthroplasties, replacement, hip':ab,ti OR 'arthroplasty, hip replacement':ab,ti OR 'hip prosthesis implantation':ab,ti OR 'hip prosthesis implantations':ab,ti OR 'implantation, hip prosthesis':ab,ti OR 'prosthesis implantation, hip':ab,ti OR 'hip replacement arthroplasty':ab,ti OR 'replacement arthroplasties, hip':ab,ti OR 'replacement arthroplasty, hip':ab,ti OR 'arthroplasties, hip replacement':ab,ti OR 'hip replacement arthroplasties':ab,ti OR 'hip replacement, total':ab,ti OR 'total hip replacement':ab,ti OR 'total hip arthroplasty':ab,ti OR 'arthroplasty, total hip':ab,ti OR 'hip arthroplasty, total':ab,ti OR 'total hip arthroplasties':ab,ti OR 'replacement, total hip':ab,ti OR 'total hip replacements':ab,ti OR tha:ab,ti)

AND

('virtual reality':ab,ti OR 'reality, virtual':ab,ti OR 'virtual reality, educational':ab,ti OR 'educational virtual realities':ab,ti OR 'educational virtual reality':ab,ti OR 'reality, educational virtual':ab,ti OR 'virtual realities, educational':ab,ti OR 'virtual reality, instructional':ab,ti OR 'instructional virtual realities':ab,ti OR 'instructional virtual reality':ab,ti OR 'realities, instructional virtual':ab,ti OR 'reality, instructional virtual':ab,ti OR 'virtual realities, instructional':ab,ti OR 'augmented reality':ab,ti OR 'augmented realities':ab,ti OR 'realities, augmented':ab,ti OR 'reality, augmented':ab,ti OR 'mixed reality':ab,ti OR 'mixed realities':ab,ti OR 'realities, mixed':ab,ti OR 'reality, mixed':ab,ti)

**Cochrane Central Register of Controlled Trials (CENTRAL)**

20230910

((MeSH descriptor: [Arthroplasty, Replacement, Hip] explode all trees) OR ((Arthroplasties, Replacement, Hip OR Arthroplasty, Hip Replacement OR Hip Prosthesis Implantation OR Hip Prosthesis Implantations OR Implantation, Hip Prosthesis OR Prosthesis Implantation, Hip OR Hip Replacement Arthroplasty OR Replacement Arthroplasties, Hip OR Replacement Arthroplasty, Hip OR Arthroplasties, Hip Replacement OR Hip Replacement Arthroplasties OR Hip Replacement, Total OR Total Hip Replacement OR Total Hip Arthroplasty OR Arthroplasty, Total Hip OR Hip Arthroplasty, Total OR Total Hip Arthroplasties OR Replacement, Total Hip OR Total Hip Replacements OR THA):ti,ab,kw (Word variations have been searched)))

AND

((MeSH descriptor: [Virtual Reality] explode all trees ) OR ((Reality, Virtual OR Virtual Reality, Educational OR Educational Virtual Realities OR Educational Virtual Reality OR Reality, Educational Virtual OR Virtual Realities, Educational OR Virtual Reality, Instructional OR Instructional Virtual Realities OR Instructional Virtual Reality OR Realities, Instructional Virtual OR Reality, Instructional Virtual OR Virtual Realities, Instructional):ti,ab,kw (Word variations have been searched)) OR (MeSH descriptor: [Augmented Reality] explode all trees) OR ((Augmented Realities OR Realities, Augmented OR Reality, Augmented OR Mixed Reality OR Mixed Realities OR Realities, Mixed OR Reality, Mixed):ti,ab,kw (Word variations have been searched)))

**Web of Science**

20230910

((((((((((((((((((((TS=(Arthroplasty, Replacement, Hip)) OR TS=(Arthroplasties, Replacement, Hip)) OR TS=(Arthroplasty, Hip Replacement)) OR TS=(Hip Prosthesis Implantation)) OR TS=(Hip Prosthesis Implantations)) OR TS=(Implantation, Hip Prosthesis)) OR TS=(Prosthesis Implantation, Hip)) OR TS=(Hip Replacement Arthroplasty)) OR TS=(Replacement Arthroplasties, Hip)) OR TS=(Replacement Arthroplasty, Hip)) OR TS=(Arthroplasties, Hip Replacement)) OR TS=(Hip Replacement Arthroplasties)) OR TS=(Hip Replacement, Total)) OR TS=(Total Hip Replacement)) OR TS=(Total Hip Arthroplasty)) OR TS=(Arthroplasty, Total Hip)) OR TS=(Hip Arthroplasty, Total)) OR TS=(Total Hip Arthroplasties)) OR TS=(Replacement, Total Hip)) OR TS=(Total Hip Replacements)) OR TS=(THA)

AND

(((((((((((((TS=(Virtual Reality)) OR TS=(Reality, Virtual)) OR TS=(Virtual Reality, Educational)) OR TS=(Educational Virtual Realities)) OR TS=(Educational Virtual Reality)) OR TS=(Reality, Educational Virtual)) OR TS=(Virtual Realities, Educational)) OR TS=(Virtual Reality, Instructional)) OR TS=(Instructional Virtual Realities)) OR TS=(Instructional Virtual Reality)) OR TS=(Realities, Instructional Virtual)) OR TS=(Reality, Instructional Virtual)) OR TS=(Virtual Realities, Instructional)) OR ((((((((TS=(Augmented Reality)) OR TS=(Augmented Realities)) OR TS=(Realities, Augmented)) OR TS=(Reality, Augmented)) OR TS=(Mixed Reality)) OR TS=(Mixed Realities)) OR TS=(Realities, Mixed)) OR TS=(Reality, Mixed))


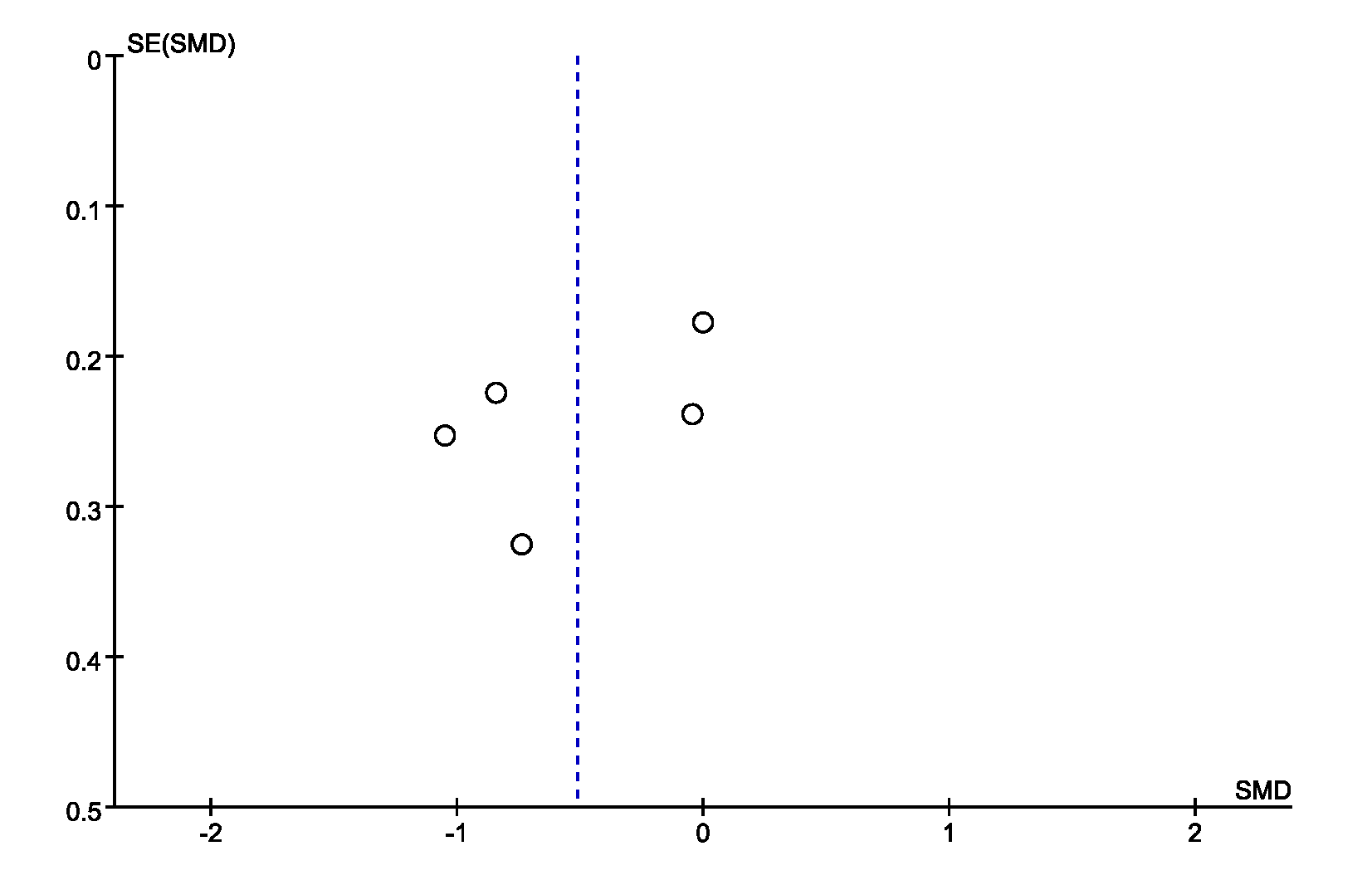


**Figure S1.** Funnel plot to assess publication bias on accuracy of inclination.


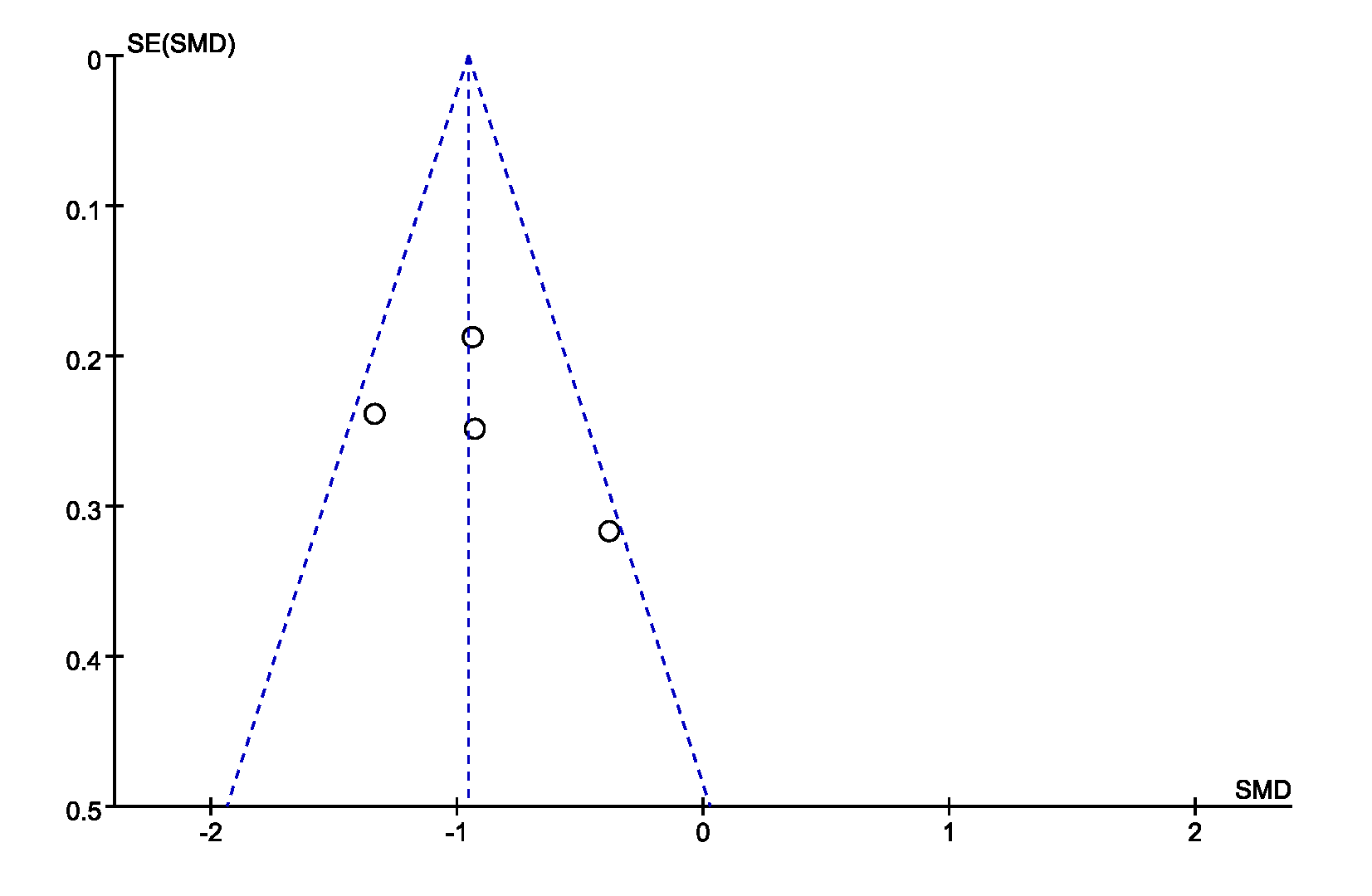


**Figure S2.** Funnel plot to assess publication bias on accuracy of anteversion.


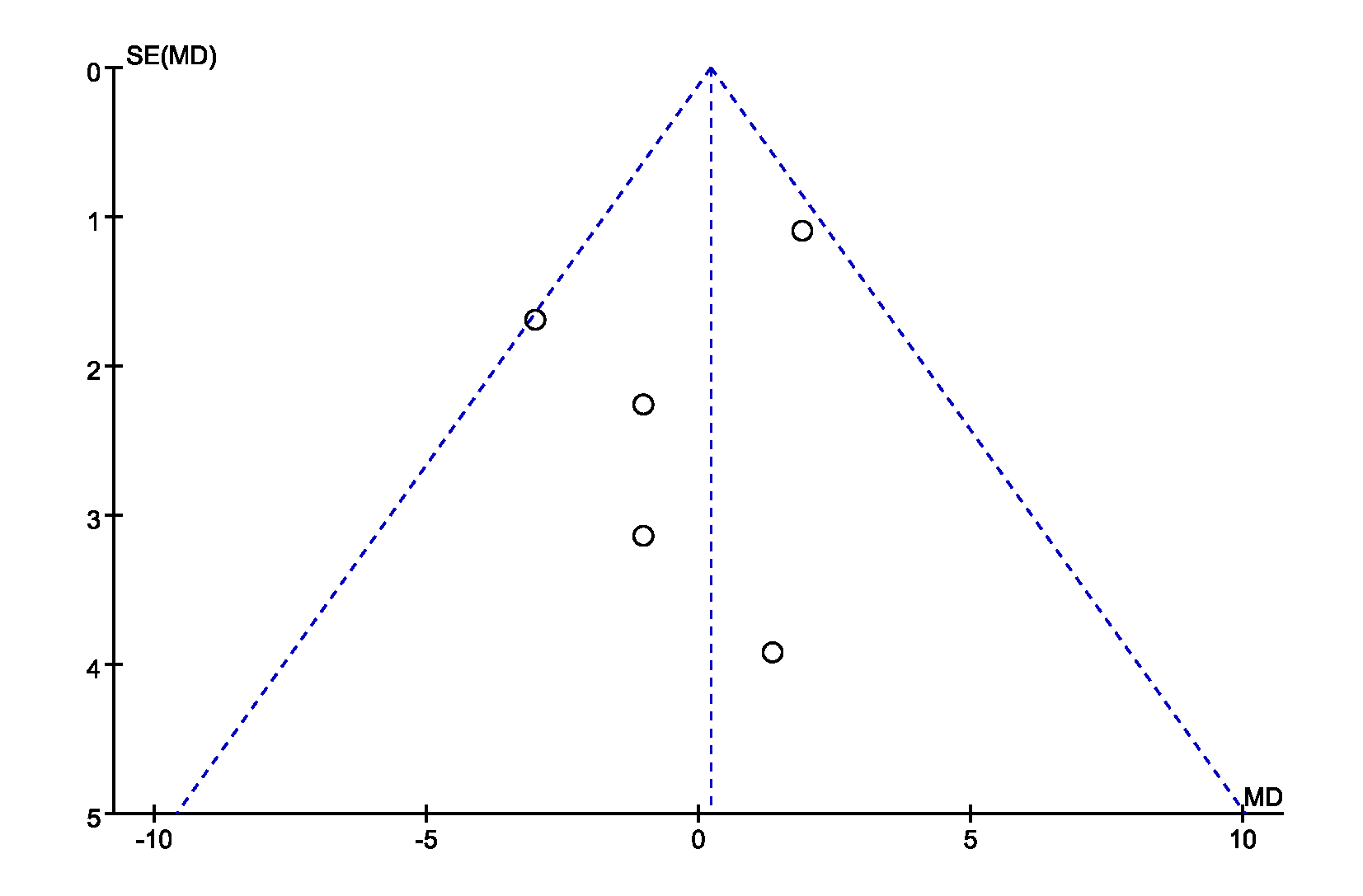


**Figure S3.** Funnel plot to assess publication bias on duration of surgery.


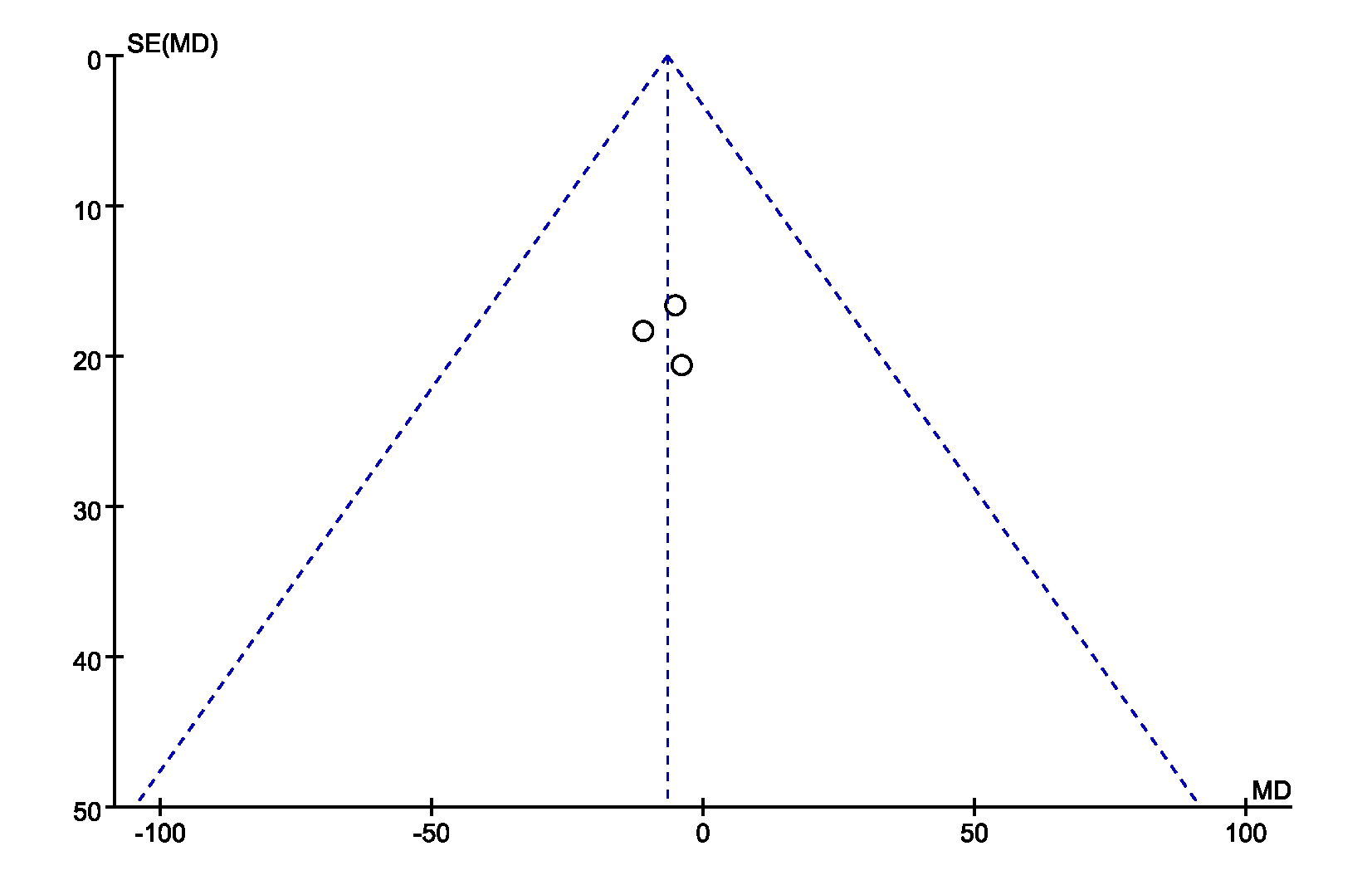


**Figure S4.** Funnel plot to assess publication bias on intraoperative blood loss.


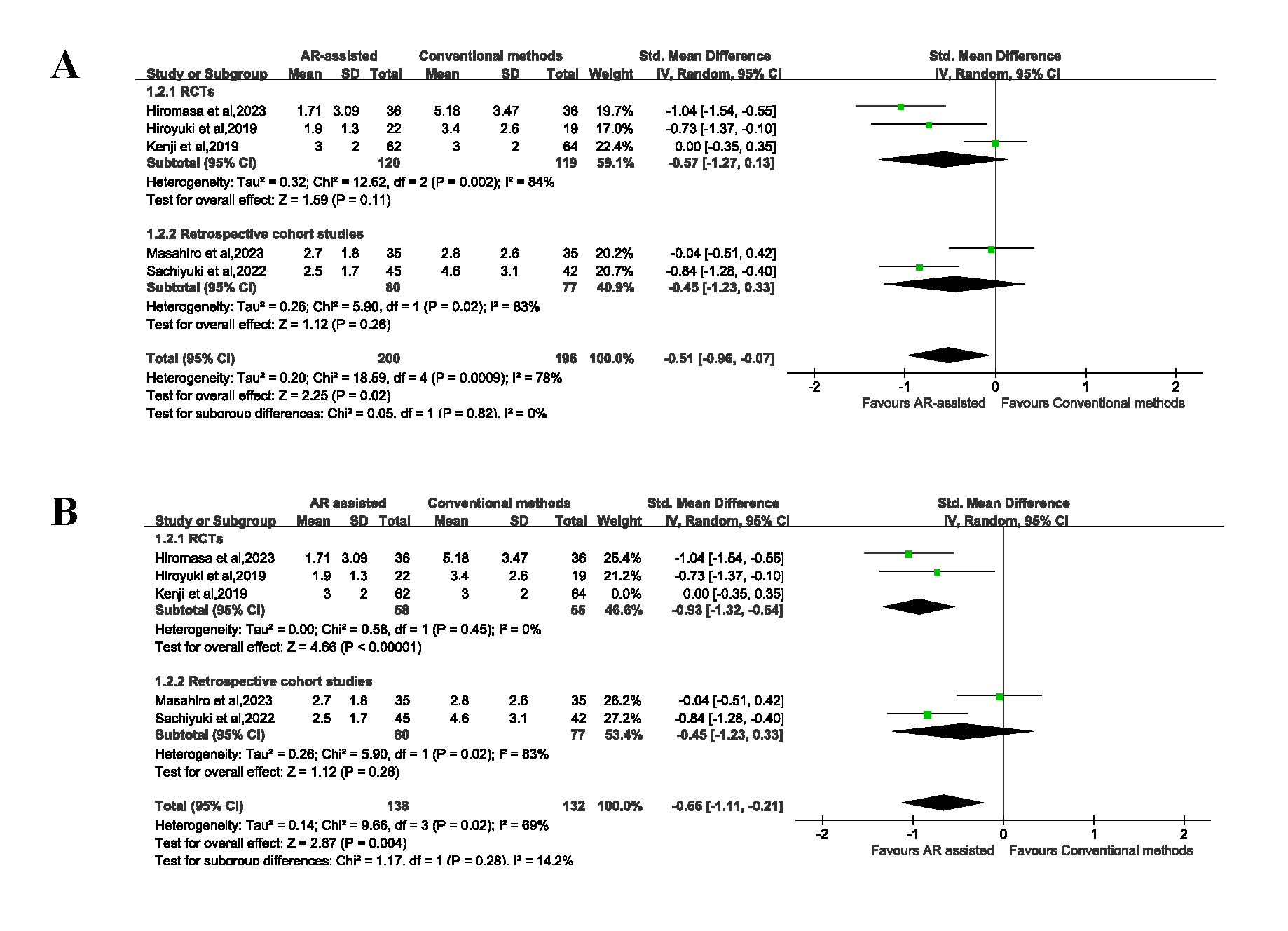


**Figure S5**. A. Forest plot of the subgroup analyses showing accuracy of inclination between two groups. B. Forest plot of the subgroup analyses showing accuracy of inclination after removing one study between two groups.


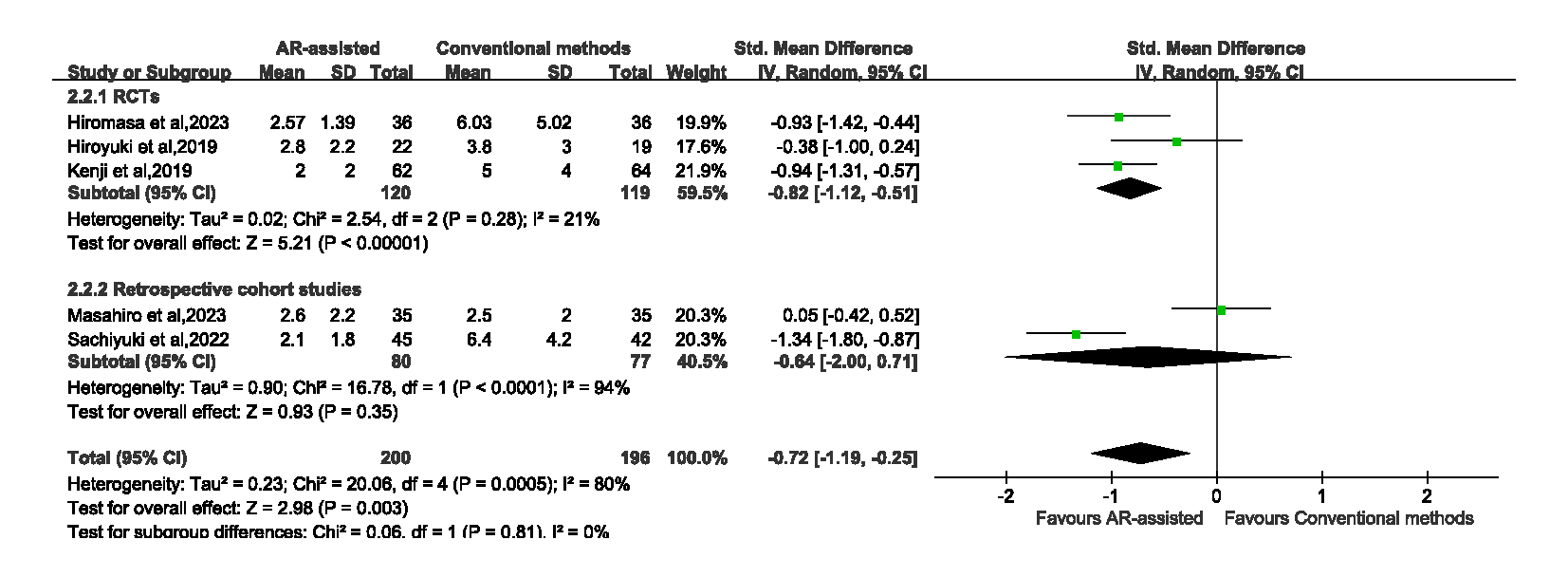


**Figure S6**. A. Forest plot of the subgroup analyses showing accuracy of anteversion between two groups.


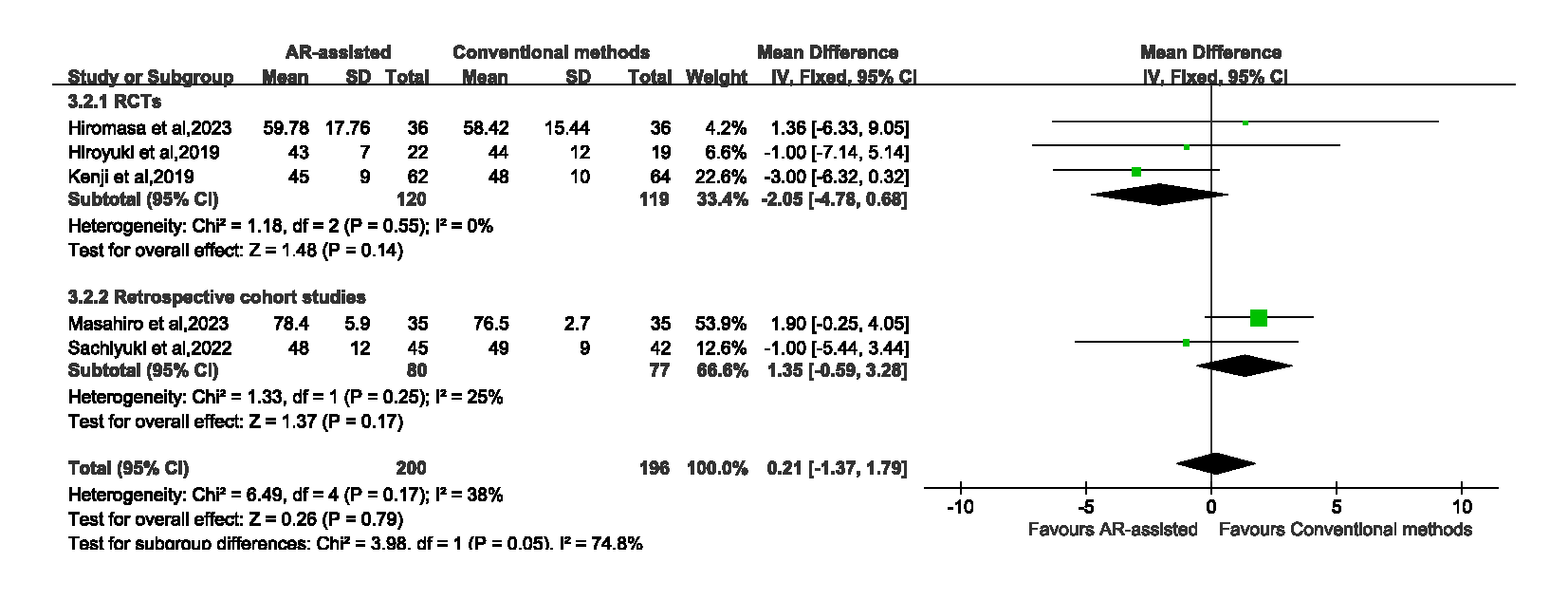


**Figure S7**. A. Forest plot of the subgroup analyses showing duration of surgery between two groups.
